# Treatment of Febrile Neutropenia and Prophylaxis in Hematologic Malignancies: A Critical Review and Update

**DOI:** 10.1155/2014/986938

**Published:** 2014-11-27

**Authors:** Paola Villafuerte-Gutierrez, Lucia Villalon, Juan E. Losa, Cesar Henriquez-Camacho

**Affiliations:** ^1^Hospital Universitario Torrejón de Ardoz, Servicio de Hematología, Calle Mateo Inurria, 28850 Madrid, Spain; ^2^Hospital Universitario Fundación Alcorcón, Servicio de Hematología, Calle Budapest 1, 28922 Madrid, Spain; ^3^Unidad de Medicina Interna, Sección de Enfermedades Infecciosas, Hospital Universitario Fundación Alcorcón, Calle Budapest 1, 28922 Madrid, Spain

## Abstract

Febrile neutropenia is one of the most serious complications in patients with haematological malignancies and chemotherapy. A prompt identification of infection and empirical antibiotic therapy can prolong survival. This paper reviews the guidelines about febrile neutropenia in the setting of hematologic malignancies, providing an overview of the definition of fever and neutropenia, and categories of risk assessment, management of infections, and prophylaxis.

## 1. Introduction

Febrile neutropenia (FN) is one of the most serious adverse events in patients with haematological malignancies and chemotherapy. Infections in neutropenic patients can rapidly progress, leading to life-threatening complications. A prompt initiation of empirical antibiotic therapy is favourable for patients with FN in order to avoid progression to sepsis and regardless of the detection of bacteraemia [1].

FN is considered a medical emergency, as infections can rapidly progress without a broad spectrum antibiotic treatment within 1 hour of fever [2]. The spectrum of bacterial pathogens isolated from FN patients has shifted from Gram-negative (1970s) species to Gram-positive organisms (since mid-1980), related to the use of antibacterial prophylaxis with fluoroquinolone and the use of indwelling catheter. Actually, the most common species isolated are Gram-positive pathogens:* Coagulase-negative staphylococci*,* Staphylococcus aureus* (including methicillin-resistant strains),* Enterococcus* spp. (including vancomycin-resistant strains),* Viridans group streptococci*,* Streptococcus pneumoniae*, and* Streptococcus pyogenes*, and drug-resistant Gram-negative pathogens:* Escherichia coli, Klebsiella *spp.,* Enterobacter* spp.,* Pseudomonas aeruginosa, Acinetobacter* spp.,* Citrobacter* spp., and* Stenotrophomonas maltophilia* [3, 4].

An overview of the risk assessment of patients with neutropenic fever and neutropenic fever syndromes and the use of empiric antibacterial and antifungal therapy for neutropenic adults will be discussed.

## 2. Definitions

Fever in neutropenic patients is classically defined as a single oral temperature of >38.3°C (101°F) [1]. Although, it is known that a neutropenic patient can be infected without fever or stay subfebrile, the definition of neutropenia is an absolute neutrophil count (ANC) < 1500 cells/microL, and severe neutropenia is usually defined as an ANC < 500 cells/microL or that is expected to decrease below 500 cells/microL during the next 48 hours, and profound neutropenia is an ANC < 100 cells/microL [1]. The risk of clinically important infection rises as the neutrophil count falls below 500 cells/microL and is higher in those with a prolonged duration of neutropenia (>7 days).

## 3. Risk of Complications

Patients who develop neutropenia can be categorized as at low risk or high risk of complications and thus poor outcome. The risk assessment has practical implications to dictate the management (including the need for inpatient admission, choice of antibiotics, and prolonged hospitalization).

Some guidelines (ESMO [5], ASCO [6], and NCCN [7]) recommend the use of the Multinational Association for Supportive Care in Cancer (MASCC) index to identify patients at low risk of complications to be treated as outpatients (see Table 1) [8]. In the MASCC study, factors associated with good prognosis in cancer patients were burden of the illness (mild or moderate clinical symptoms at presentation): absence of hypotension; absence of chronic obstructive pulmonary disease; presence of solid tumour or, in patients with hematologic malignancies, absence of previous fungal infection; outpatient status, absence of dehydration; and age lower than 60 years. A patient with a MASCC score > 21 points is considered “low risk” with positive and negative predictive values of 91% and 36%, respectively. Those patients can be treated using oral antibiotics. The IDSA guideline favours the expert clinical criteria and considers low-risk patients as those who are expected to be neutropenic (ANC < 500 cells/microL) for ≤7 days and those who have no active comorbidities or evidence of significant hepatic or renal dysfunction. Similarly, high-risk patients are those who are expected to be neutropenic (ANC < 500 cells/microL) for >7 days. Patients with neutropenic fever who have on-going comorbidities or evidence of significant hepatic or renal dysfunction are also considered to be high risk, regardless of the duration of neutropenia. The NCCN guidelines consider other risk factors but they also support the use of the MASCC index [7] (see Table 2). ASCO guideline presents a list of conditions which makes the outpatients high risk without considering the MASCC score [6].

## 4. Antibiotic Prophylaxis

Bacterial infections are a major cause of morbidity and mortality in patients who are neutropenic following chemotherapy for malignancy [9]. Trials have shown the efficacy of antibiotic prophylaxis in reducing the incidence of bacterial infections [10]. The IDSA [1], ESMO [5], ASCO [6], and NCCN [7] recommend antibacterial prophylaxis with a fluoroquinolone for high-risk patients (who are going to be neutropenic for >7 days), although the Australian Consensus Guidelines consider that the evidence was not strong enough to recommend antibiotic prophylaxis, except for stem cell transplantation patients and palliative patients with BM failure [11].

Meta-analyses have indicated that antibiotic prophylaxis with fluoroquinolone may reduce the overall mortality in neutropenic patients of an intermediate- to high-risk group as well as the incidence of fever and bacteraemia [12, 13]. A Cochrane review (109 trials, 13579 participants) showed that prophylaxis significantly reduced the risk of death from all causes (RR: 0.66, 95% CI 0.55 to 0.79), the risk of infection-related death (RR 0.61, 95% CI 0.48 to 0.77), the occurrence of fever (RR 0.80, 95% CI 0.74 to 0.87), and clinically documented infection (RR 0.65, 95% CI 0.56 to 0.76). There were no significant differences between fluoroquinolone prophylaxis and TMP-SMX prophylaxis with regard to death from all causes or infection; however, fluoroquinolone prophylaxis was associated with fewer side effects leading to discontinuation. Antibiotic prophylaxis in afebrile neutropenic patients significantly reduced all-cause mortality [14].

Some controversy remains regarding precisely which patient groups are the most appropriate candidates for fluoroquinolone prophylaxis. Some randomized trial did not include allogeneic HSCT recipients. Accordingly, many experts do not recommend fluoroquinolone prophylaxis for neutropenic autologous HSCT recipients [1].

Another quinolone, moxifloxacin, has been used as antibacterial prophylaxis for autologous HSCT, showing in a small double blind and placebo controlled randomized clinical trial that is superior to prevent bacteraemia and shortened febrile episodes. No significant increase of adverse events in the moxifloxacin arm was observed (adverse events reported were diarrhoea,* C. difficile* associated diarrhoea, exanthema, and QT prolongation) [15].

Although fluoroquinolone agents are widely used for prevention and management of infections in neutropenic patients, there is a main concern about the emergence of fluoroquinolone-resistant bacteria [16]. Fluoroquinolone resistance is linked to community fluoroquinolone consumption and the prophylaxis efficacy is reduced when the prevalence of fluoroquinolone Gram-negative bacillary resistance exceeds 20% [17]. The emergence of methicillin-resistant* Staphylococcus aureus* (MRSA) is associated with the use of multiple antibiotics, particularly with fluoroquinolones. The same occurs with the colonization by* C. difficile* and vancomycin-resistant enterococci (VRE) [17, 18].

In contrast, some cohort studies show that quinolone prophylaxis did not affect the emergence of quinolone-resistant Gram-negative isolates from blood cultures in FN patients. Probably, the resistance is not induced by quinolone alone [19, 20].

Finally, the antibiotic prophylaxis is effective and the emergence of resistant bacteria should be continuously monitored for the detection of local antibiotic resistance bacteria and discriminate the appropriate antibiotic that can be used based on different settings.

## 5. Antifungal Prophylaxis 

Invasive fungal infections produced by yeasts and molds are the main infectious cause of mortality in patients with haematological malignancies. In the era preantifungal prophylaxis,* Candida* spp. accounted for the majority of fungal infections that occurred during neutropenia, followed by* Aspergillus* spp. Actually,* Aspergillus* has surpassed* Candida* as a cause of invasive fungal infections due to the use of antifungal prophylaxis against* Candida* spp. [21].

### 5.1. Candida Infection

Meta-analyses and randomized trials have determined that fluconazole is efficacious in preventing* Candida* infections in high-risk patients [22, 23]. Among lower-risk patient populations, invasive candidiasis is rare and generally does not merit routine fluconazole prophylaxis.

### 5.2. Aspergillus Infection

The efficacy of antifungal agents with activity against* Aspergillus* spp. and other molds (voriconazole, posaconazole, amphotericin B) has been evaluated, suggesting that prophylaxis prevents invasive fungal infections. Cornely et al. showed that prophylaxis with Posaconazole was superior to prophylaxis with fluconazole or itraconazole in the prevention of invasive fungal infection and resulted in lower mortality in patients with acute myelogenous leukemia or myelodysplastic syndromes who are undergoing remission-induction chemotherapy [24]. Aspergillus prophylaxis with posaconazole shows benefit in patients with graft-versus-host disease (GVHD) who were receiving immunosuppressive therapy [25]. All of the agents with activity against molds also have activity against* Candida* spp.

Fluconazole was the first azole used for antifungal prophylaxis, having high systemic activity, excellent tolerability, low toxicity, and cheap generic formulations and prevents infection with all species of Candida except for* C. krusei* or* C. glabrata* [26]. It is important to note that Fluconazole has no activity against* Aspergillus* or other molds.

Other agents such as itraconazole, voriconazole, posaconazole, and caspofungin are all acceptable alternatives [1]. The other echinocandins (anidulafungin and micafungin) have not been studied specifically for empiric antifungal therapy; however, NCCN panel members consider them to likely be effective, based on the data for caspofungin [7].

The echinocandins have a broader spectrum of activity than fluconazole and an excellent safety record. Micafungin was compared to fluconazole in a prospective, randomized, double blind comparative trial study showing that it is being superior during the neutropenic phase after haemopoietic stem cell transplant [27]. The drawback of this antifungal class is its availability only as an intravenous formulation and its high cost.

Voriconazole is available since 2003 and was initially approved for treatment of* Aspergillus* spp. infections but not against the agents of mucormycosis. Voriconazole has been best evaluated for the treatment of invasive aspergillosis and it is the preferred agent for treatment. Some trials have demonstrated its efficacy also as antifungal prophylaxis. Randomized, double blind trails compared fluconazole against voriconazole for the prevention of IFI, showing no significant difference in incidence of IFI or survival [28]. Another randomized, open-label, multicentre study comparing voriconazole and itraconazole showed no difference in terms of incidence or IFI or survival [29]. Voriconazole has both oral and IV formulations but has been noted to cause transient visual disturbances (which are not permanent or serious). A major drawback is its potential interactions with certain chemotherapy agents.

Itraconazole is active against* Aspergillus* spp. and it is available as an oral formulation. Two studies compared to fluconazole showed the prophylactic activity for fungal infections [30, 31]. A meta-analysis (including 13 randomized trials, 3597 neutropenic patients) evaluated the efficacy of itraconazole versus other forms of prophylaxis for the prevention of IFI, showing a significant reduction in the incidence of IFI, of invasive yeast infections and mortality [32]. A major drawback is its potential interactions with certain chemotherapy agents (best documented with cyclophosphamide and vinca alkaloids).

Posaconazole is available as an oral suspension since 2007. Recently, the FDA has approved the intravenous presentation of posaconazole. It is active against* Candida* spp.,* Aspergillus* spp.,* Zygomycetes,* and* Fusarium*. A randomized, multicentre single-blind study evaluated the efficacy and safety of posaconazole compared to fluconazole or itraconazole as prophylaxis for each cycle of chemotherapy in patients with AML or myelodysplastic syndrome and prolonged neutropenia. Posaconazole was superior over standard triazoles in preventing IFIs and significantly better in preventing invasive aspergillosis. Survival was significantly longer among recipients of posaconazole than among recipients of fluconazole or itraconazole [24]. Another randomized double-blind trial found no difference between posaconazole and fluconazole to prevent IFIs but posaconazole was superior to fluconazole preventing invasive aspergillosis. Mortality was similar between both groups but the number of deaths from IFI was lower in posaconazole group [25].

Its major drawbacks of oral suspension of posaconazole include variability of blood levels after oral admission. It should be taken with a full meal or with liquid nutritional supplements or an acidic carbonated beverage. If the patient is not eating, absorption is greatly impeded. In that case, the delayed-release tablets result in higher plasma drug concentrations than the oral suspension regardless of food intake. Patients who are unable to take medications orally or who are expected not to absorb oral medications should be given IV posaconazole.

The other disadvantage is its potential interactions with certain chemotherapy agents, such as cyclophosphamide, and the vinca alkaloids, such as vincristine. The NCCN Guidelines panel advises that prophylaxis with posaconazole, itraconazole and voriconazole should be avoided in patients receiving vinca alkaloid-based regimens (such as vincristine in acute lymphoblastic leukemia) because of the potential of these azoles to inhibit the cytochrome P3A4 isoenzyme reducing clearance of vinca alkaloids [7].

Finally, it is important to note that antifungal prophylaxis could decrease the sensitivity of biomarkers such as galactomannan [33]. Theoretically, the use of prophylaxis could impact the choice of the strategy during the neutropenia but few data are available [34].

## 6. When to Start Antibiotics?

ESMO, IDSA, and ASCO guidelines recommend antibiotic prophylaxis with a fluoroquinolone for patients who are going to be neutropenic for >7 days [1, 5, 6].

In all febrile neutropenic patients, empiric broad-spectrum antibacterial therapy should be initiated immediately after blood cultures have been obtained and before any other investigations have been completed [35]. Antimicrobial therapy should be administered within 60 minutes of presentation [36]. Mortality in neutropenic patients with Gram-negative bacteraemia can approach 40%, if an empirical treatment is not promptly undertaken [37].

The specific empirical regimen remains controversial. Actual guidelines recommend, for high-risk patients, starting monotherapy with a beta-lactam with activity against* Pseudomonas aeruginosa *(piperacillin-tazobactam, meropenem, imipenem-cilastatin, ceftazidime, and cefepime) [38]. Regarding the choice of beta-lactam, no single agent is clearly superior, although, in a meta-analysis (44 trials included), mortality was significantly lower with piperacillin-tazobactam compared to other antibiotics (RR 0.56, 95% CI 0.34 to 0.92, 8 trials, 1314 participants), without heterogeneity. Carbapenems resulted in a higher rate of antibiotic-associated and* Clostridium difficile*-associated diarrhoea, and the all-cause mortality was higher with cefepime compared to other beta-lactams [38]. Concerns regarding increased overall mortality with cefepime have been largely dismissed by the IDSA and ASCO after a reanalysis of the cefepime data by the FDA [39].

A two-drug regimen can be chosen in patients suspected of infection caused by resistant Gram-negative. Thus, a second gram-negative antibiotic should be added. Although a recent meta-analysis (seventy-one trials published between 1983 and 2012) showed that beta-lactam monotherapy is advantageous compared with beta-lactam-aminoglycoside combination therapy with regard to survival (RR 0.80, 95% CI 0.64 to 0.99), adverse events (numbers needed to harm 4; 95% CI 4 to 5), and fungal superinfections (RR 0.70, 95%CI 0.49 to 1.00) [40] (see Tables 3 and 4).

All the guidelines recommend not including vancomycin routinely in the initial regimen and not adding it empirically for persistent fever, although the IDSA guideline strongly recommends adding vancomycin in cases of hemodynamic instability, pneumonia, clinically evident catheter-related infection, skin and soft tissue infections, severe mucositis when fluoroquinolone prophylaxis has been used and ceftazidime is used empirically, and known colonization with methicillin-resistant* Staphylococcus aureus* [1].

For low risk patients eligible for outpatient management, the regimen of choice is the combination of fluoroquinolone and amoxicillin-clavulanic acid (or clindamycin for penicillin-allergic patients) as long as no fluoroquinolone prophylaxis was used, the patient tolerates oral medication, and the rate of resistance to fluoroquinolone is less than 20% [6]. Ciprofloxacin should not be employed as a solo agent because of its poor coverage of Gram-positive organisms [1]. When a fluoroquinolone cannot be used, a broad-spectrum beta-lactam active against* Pseudomonas *and suitable for outpatient use should be used. Recently, a randomized, double-blind, multicenter clinical trial (intention to treat analysis: 169 patients in the moxifloxacin group versus 164 patients in the combination therapy) using a single daily oral dose of Moxifloxacin 400 mg in low-risk febrile neutropenic patients (MASCC score > 20) compared with ciprofloxacin (750 mg every 12 hours) plus amoxicillin/clavulanic acid (1000 mg every 12 hours) showed similar efficacy and safety (80% versus 82%, resp.). The most common adverse event was diarrhea in the combination therapy arm (42 versus 21 patients), but more neurologic events (eg, dizziness, vertigo, sleep disorder) were seen in the moxifloxacin arm [41].

If an infectious source of fever is identified, antibiotics should be continued for at least the standard duration indicated for the specific infection (e.g., 14 days for* Escherichia coli* bacteraemia); antibiotics should also continue at least until the absolute neutrophil count (ANC) is ≥500 cells/microL or longer if clinically indicated [1]. In case of no source of infection is identified and cultures are negative, the timing of discontinuation of antibiotics is usually dependent on resolution of fever and clear evidence of bone marrow recovery. If the patient has been a febrile for at least two days and the ANC is >500 cells/microL and is showing a consistent increasing trend, antibiotics may be stopped [1]. If the patient is still neutropenic and antibiotic therapy is stopped, the patient should be kept hospitalized under close observation for at least 24–48 hours. If fever recurs, antibiotics should be restarted urgently after obtaining blood cultures and performing other relevant evaluation based on clinical judgment [42].

## 7. Persistent Fever

Persistent fever is an episode of fever during neutropenia that does not resolve after 5 days of broad-spectrum antibacterial agents. The median time to defervescence following the initiation of empiric antibiotics in patients with hematologic malignancies is five days, in contrast with only two days for patients with solid tumours [1]. Modification of the initial antibacterial therapy is not needed for persistent fever alone if the patient is in good clinical condition. In that case, the best clinical option should be watchful waiting. However, patients who remain febrile after the initiation of empiric antibiotics should be revaluated for possible infectious sources.

Management algorithms have been developed for the reassessment of neutropenic patients with persistent fever after two to four days and after four or more days [1]. Consideration should be given to invasive fungal infection identified as a common cause of persistent fever in neutropenic patients [43]. To avoid the onset of invasive fungal infection in neutropenic patients, three approaches have been developed, which are often combined: antifungal prophylaxis, empirical antifungal therapy, and preemptive antifungal approaches. Before efficient antifungal prophylaxis was available and before indirect biological markers and effective imaging were assessed, the only acceptable approach was empirical antifungal therapy in patients with persistent or recurrent unexplained fever refractory to broad-spectrum antibiotics [34].

Based on trials adding amphotericin B after 4 to 7 days of persistent fever, [44, 45] the guidelines recommend adding an empiric antifungal agent after four to seven days in high-risk neutropenic patients who are expected to have a total duration of neutropenia >7 days who have persistent or recurrent fever and in whom reassessment does not yield a cause [1]. Additionally, sepsis status (severe sepsis or septic shock), focused infection (lung, central nervous system, sinus, abdominal, or skin), and clinical judgment can be used to decide empiric antifungal therapy and avoid unnecessary treatment [46].

The incidence of fungal infection (especially those caused by* Candida* or* Aspergillus* spp.) rises after patients have experienced more than seven days of persistent neutropenic fever [47]. In patients who are clinically unstable or have a suspected fungal infection, antifungal therapy should be considered even earlier than what is recommended for empiric therapy.

The choice of agent for empiric antifungal therapy depends upon which fungi are most likely to be causing infection, as well as the toxicity profiles and cost [1]. The IDSA guideline for empiric antifungal therapy recommends lipid formulation of amphotericin B, caspofungin, voriconazole, or itraconazole as suitable options for empiric antifungal therapy in neutropenic patients [1, 48–50] (see Table 5).

For persistently febrile patients with pulmonary nodules or nodular pulmonary infiltrates, invasive mold infection should be strongly suspected, and prompt assessment frequently requires bronchoscopy with bronchoalveolar lavage with cultures, stains, and* Aspergillus* galactomannan antigen testing to distinguish bacterial from mold pathogens, while simultaneously initiating antibacterial and antimold therapy until the specific aetiology is established. Voriconazole or a lipid formulation of amphotericin B is the drug of choices for invasive mold infection. Caspofungin is not preferred because of high failure rates in preventing and treating invasive aspergillosis. If mucormycosis is suspected, an amphotericin B formulation should be given since voriconazole has no activity against* Mucor* species [51] There is insufficient evidence to conclusively determine the superiority of any agent; the choice of the initial antifungal agent may vary based on epidemiology and local susceptibility patterns. IDSA guideline recommends a diagnostic imaging workup (chest and/or sinus computed tomography) to rule out fungal infections in patients with neutropenia expected to last >7 days and persistent fever [1].

In case of febrile neutropenic patients who have been receiving antimold prophylaxis, a different class of antifungal agent with activity against molds should be used for empiric therapy. Finally, caspofungin and other echinocandins are not active against* Cryptococcus* spp.,* Trichosporon* spp., and filamentous molds other than* Aspergillus* spp., such as* Fusarium* spp. In addition, some yeast can demonstrate relative resistance to these drugs (*C. parapsilosis*,* C. rugosa*,* C. guilliermondii*, and noncandidal yeasts). Moreover, the clinical efficacy of the echinocandins for endemic fungi (*Histoplasma*,* Blastomyces*,* Coccidioides* spp.) has not been demonstrated.

## 8. Preemptive Antifungal Therapy

In recent years, some authors have suggested that limiting antifungal therapy to selected patients may reduce the perceived unnecessary use of overempirical antifungal treatment, reduce toxicity, and reduce costs without increasing IFI-related mortality [52, 53].

There is no consensual definition of a preemptive therapy, but the common goal is to use the current screening tests (serum galactomannan, beta-D-glucan assay, and high-resolution chest CT) to postpone starting antifungal therapy until IFI is more likely. This approach is best suited for patients receiving prophylaxis with an antiyeast agent, such as fluconazole, where the concern is mainly mold pathogens and broadening the coverage to include antimold agents is appropriate.

In 2005, Maertens et al. evaluated the feasibility of a “preemptive” approach based on the incorporation of sensitive, noninvasive diagnostic tests (galactomannan and CT-scanning) for high-risk neutropenic patients who had received fluconazole prophylaxis while avoiding empirical therapy. This approach reduced the rate of antifungal use for FN from 35% to 7.7%, lowering the exposure to expensive and potentially toxic drugs and led to the early initiation of antifungal therapy in about 7% of episodes that had not been clinically suspected of being related to an invasive fungal disease [54].

In 2009, Cordonnier et al. published a randomized open label trial comparing an empirical antifungal strategy with a preemptive one in high-risk neutropenic patients using a galactomannan and a chest CT. This trial showed that preemptive treatment increased the incidence of invasive fungal disease, without increasing mortality, and decreased the costs of antifungal drugs but empirical treatment showed better survival rates for patients receiving induction chemotherapy [55].

Girmenia et al. showed that an intensive clinically driven diagnostic strategy based on galactomannan tests and CT scans in selected patients with neutropenic fever reduced the use of antifungal treatment by 43% compared to that used with a standard empirical approach. At the 3-month follow-up, 63% of the patients with invasive fungal disease had survived, and no cases of undetected invasive fungal disease were found [56].

Controversy about the reduction of antifungal consumption by the preemptive strategy is not resolved. The larger observational study, including 190 patients treated with empirical antifungal therapy (neutropenic patients with fever without known source of infection and unresponsive to antibacterial agents) and 207 with preemptive antifungal therapy (patients with laboratory tests or radiographic signs indicative of invasive fungal disease, without culture or histology proof) published by Pagano et al., showed that the rate of invasive fungal disease was higher in the preemptive antifungal therapy (23.7% versus 7.4%, *P* < 0.001) as well as the overall mortality rates (15.9% versus 6.3%, *P* = 0.002) [57]. Of note, the definition for an early preemptive therapy, in the included population, was not used (screening tests to postpone starting antifungal therapy until IFI is more likely).

Recently, Morrissey et al. in an open label randomized controlled trial (240 patients), compared an empirical strategy (culture and histology), with a preemptive approach using twice-weekly blood testing with galactomannan and PCR to detect* Aspergillus* spp. A CT scan was performed in the case of positive biomarker(s) or of persistent fever. The use of empirical antifungal drugs was significantly lower in the preemptive compared with the empirical group (15% versus 32%; *P* = 0.002). Overall survival was not different between groups. IFD were significantly more frequent in the preemptive group than in the standard group (24.5% versus 4.1%; *P* < 0.0001) [58].

Despite the risk of overtreatment in patients who do not have an invasive fungal disease, the empirical approach seems able to guarantee a better outcome in hematologic patients, remains easy, reproducible, safe, and cheap in terms of diagnostic methods, probably making it the best choice when adequate microbiological and radiological support is lacking and neutropenia lasts more than 10–15 days. For neutropenia of shorter duration (<10 days), the benefit of both strategies is similar and both are even debatable considering the low risk of IFD in that setting [55].

## 9. Catheter Removal

Central venous catheter- (CVC-) related infections are common in patients with neutropenic fever. Differential time to positivity 120 min of qualitative blood cultures performed on specimens simultaneously drawn from the CVC and a vein suggests a central line associated bacteraemia. In addition to 14 days of systemic antibiotics, CVC removal is recommended in which any of the following organisms is implicated:* S. aureus*,* P. aeruginosa*,* Candida* spp., other fungi, and rapidly growing nontuberculous mycobacteria. This recommendation is based upon observational studies showing improved clearance of infection among patients with* S. aureus*,* P. aeruginosa*, or* Candida* spp. bloodstream infections in which the CVC was removed [59, 60]. In a study of cancer patients with bacteraemia caused by rapidly growing mycobacteria, CVC removal was associated with a significantly reduced rate of relapse of bacteraemia [61].

Catheter removal is also recommended for tunnel infection, port pocket infection, septic thrombosis, endocarditis, sepsis with hemodynamic instability, and bloodstream infection that persists despite ≥72 hours of therapy with appropriate antibiotics, even when pathogens other than those described above are isolated [1]. Prolonged treatment (4–6 weeks) is recommended for complicated infection, defined as the presence of deep tissue infection, endocarditis, septic thrombosis, or persistent bacteraemia or fungemia occurring 72 h after catheter removal in a patient who has received appropriate antimicrobials.

For CVC-associated bacteraemia caused by coagulase-negative staphylococci, the CVC may be retained if the patient is stable, using systemic therapy with or without antibiotic lock therapy [1, 5].

## 10. Conclusion

(i) FN is a medical emergency with high mortality without an appropriate treatment. It is imperative to assess the risk for serious complications in neutropenic patients to decide the use of prophylaxis and an antimicrobial therapy and the need for inpatient admission.

(ii) Low-risk patients with FN are those in whom the duration of neutropenia (ANC < 500 cells/microL) is expected to be ≤7 days and those with no comorbidities. Those patients can be treated as outpatients.

(iii) High-risk patients with FN are those who are expected to be neutropenic (ANC < 500 cells/microL) for >7 days and those with comorbidities. Those patients should be admitted to hospitalization.

(iv) Blood stream infection is a serious complication in neutropenic patients. Gram-positive bacteria are the most common causes of infection, but drug-resistant Gram-negative bacteria are generally associated with the most serious infections.

(v) Prophylaxis is not necessary in all patients and should only be used in high-risk patients to avoid the emergence of resistant pathogens.

(vi) Empiric broad-spectrum antibacterial therapy should be initiated immediately after blood cultures have been obtained in high-risk patients with FN. Empiric antibacterial therapy should be started within 60 minutes of presentation in all patients presenting with neutropenic fever. Preemptive antifungal therapy strategy seems to be similar to empirical approach in low-risk patients with FN.

(vii) Fungal pathogens are more common in high-risk patients.* Candida *spp. and* Aspergillus *spp. account for the most invasive fungal infections during neutropenia.

(viii) Selecting antimicrobial agents for prophylaxis and/or empirical therapy should be based on the local susceptibility and resistance patterns of microorganisms.

## Figures and Tables

**Table 1:** MASCC score [8].

Clinical parameters	Score^*^
Burden of illness: no or mild symptoms^†^	5
No hypotension	5
No chronic obstructive pulmonary disease^‡^	4
Solid tumour or no previous fungal infection^§^	4
No dehydration	3
Outpatient status	3
Burden of illness: moderate symptoms	3
Patient's age < 60 years	2

MASCC: Multinational Association for Supportive Care in Cancer.

Scores > 21 indicate a low risk for medical complications.

^*^The maximum theoretical score is 26.

^†^Burden of FN refers to the general clinical status of the patient as influenced by the febrile neutropenic episode. It should be evaluated on the following scale: no or mild symptoms (score of 5), moderate symptoms (score of 3), and severe symptoms or moribund (score of 0). Scores of 3 and 5 are not cumulative.

^‡^Chronic obstructive pulmonary disease means active chronic bronchitis, emphysema, decrease in forced expiratory volumes, or need for oxygen therapy and/or steroids and/or bronchodilators requiring treatment at the presentation of the febrile neutropenic episode.

^§^Previous fungal infection means demonstrated fungal infection or empirically treated suspected fungal infection.

**Table 2:** Initial risk assessment for febrile neutropenic patients (adapted from NCCN guideline) [7].

Low risk (score > 21 on the MASCC risk score) OR:	High risk (score < 21 on the MASCC risk score) OR:
Outpatient status at time of development of fever	Inpatient status at time of development of fever
No associated acute comorbid illness	Significant medical comorbidity or clinically unstable
Anticipated short duration of severe neutropenia (less than 7 days)	Anticipated prolonged severe neutropenia (ANC < 100 cells and >7 days)
Good performance status (ECOG 01)	Hepatic insufficiency
No hepatic insufficiency	Renal insufficiency
No renal insufficiency	Pneumonia or other complex infections at clinical presentation
	Alemtuzumab
	Mucositis grade 3-4

**Table 3:** Intravenous antibiotics for empirical therapy of fever in neutropenic patients.

Monotherapy	Two-drug regimen
Piperacillin-tazobactam	Piperacillin-tazobactam + amikacin
Imipenem-cilastatin	Imipenem-cilastatin + amikacin
Meropenem	Meropenem + amikacin
Ceftazidime	Ceftazidime + amikacin
Cefepime	Ceftriaxone + amikacin

**Table 4:** Dosages of administrations of intravenous antibiotics for empirical treatment of febrile neutropenia.

Antibiotics	Doses
Amikacin	15–20 mg/kg every 24 h
Gentamicin	5–7 mg/kg every 24 h
Tobramycin	5–7 mg/kg every 24 h
Piperacillin-tazobactam	3.375 g/500 mg every 8 h or every 6 h
Ceftazidime	2 g every 8 h
Cefepime	2 g every 8 h
Imipenem-cilastatin	1 g every 8 h or every 6 h
Meropenem	1-2 g every 8 h
Vancomycin	15–20 mg/kg every 12 h
Linezolid	600 mg every 12 h
Daptomycin	6 mg/kg every 24 h
Teicoplanin	0.4–1.2 g qid (2 doses within the first 24 hours)
Ciprofloxacin	400 mg every 8 h or every 12 h
Levofloxacin	500–750 mg every 24 h

**Table 5:** Dosages of administrations of antifungal agents for empirical treatment of febrile neutropenia.

Antifungal	Doses
Fluconazole	400 mg/24 h IV/PO
Itraconazole	400 mg/24 h PO
Voriconazole	6 mg/kg every 12 h × 2 doses, then 4 mg/kg every 12 h; 200 mg/12 h PO
Posaconazole	Prophylaxis: 200 mg PO every 8 h
Caspofungin	70 mg IV initial doses, then 50 mg/24 h IV
Micafungin	100 mg/24 h IV for candidemia and 50 mg/24 h IV as prophylaxis; 150 mg/24 h IV for *Aspergillus* spp. infection
Anidulafungin	200 mg IV initial doses, then 100 mg/24 h IV
