# Corrigendum to “Treatment of Febrile Neutropenia and Prophylaxis in Hematologic Malignancies: A Critical Review and Update”

**DOI:** 10.1155/2019/4120631

**Published:** 2019-07-16

**Authors:** Paola Villafuerte-Gutierrez, Lucia Villalon, Juan E. Losa, Cesar Henriquez-Camacho

**Affiliations:** ^1^Hospital Universitario Torrejón de Ardoz, Servicio de Hematología, Calle Mateo Inurria, 28850 Madrid, Spain; ^2^Hospital Universitario Fundación Alcorcón, Servicio de Hematología, Calle Budapest 1, 28922 Madrid, Spain; ^3^Unidad de Medicina Interna, Sección de Enfermedades Infecciosas, Hospital Universitario Fundación Alcorcón, Calle Budapest 1, 28922 Madrid, Spain

The article titled “Treatment of Febrile Neutropenia and Prophylaxis in Hematologic Malignancies: A Critical Review and Update” [1] was found to contain material from previously published work. The articles are as follows:

(i) Freifeld, A. G., E. J. Bow, K. A. Sepkowitz, M. J. Boeckh, J. I. Ito, C. A. Mullen, I. I. Raad, K. V. Rolston, J.-A. H. Young, and J. R. Wingard. “Clinical Practice Guideline for the Use of Antimicrobial Agents in Neutropenic Patients with Cancer: 2010 Update by the Infectious Diseases Society of America”, Clinical Infectious Diseases, 2011. https://doi.org/10.1093/cid/cir073. [2] (Cited as reference [1])

(ii) J. Gea-Banacloche, “Evidence-based approach to treatment of febrile neutropenia in hematologic malignancies,” Hematology/the Education Program of the American Society of Hematology, vol. 2013, no. 1, pp. 414–422, 2013. [3] (Cited as reference [39])

(iii) Amar Safdar, “Principles and Practice of Cancer Infectious Diseases,” 2011. https://doi.org/10.1007/978-1-60761-644-3. [4] (Not cited)

(iv) Aarti S. Bhardwaj & Shyamala C. Navada (2013) Management of Chemotherapy-Induced Neutropenic Fever, Hospital Practice, 41:1, 96-108, DOI: 10.3810/hp.2013.02.1015. [5] (Not cited)

(v) L. Pagano, M. Caira, A. Nosari et al., “The use and efficacy of empirical versus pre-emptive therapy in the management of fungal infections: the HEMA e-Chart project,” Haematologica, vol. 96, no. 9, pp. 1366–1370, 2011. Doi:10.3324/haematol.2011.042598. [6] (Cited as reference [57])

The article [1] includes an overlap of 437 words with Freifeld, A. G. et al. [2], 405 words with J. Gea-Banacloche et al. [3], 342 words with Amar Safdar et al. [4], 252 words with Aarti S. Bhardwaj et al. [5], and 221 words with L. Pagano et al. [6].

The authors said they did not intend to copy the work of others, but this occurred because some phrases are difficult to rephrase and no new knowledge was presented in this article. The authors apologized for this matter.

## References

[1] Villafuerte-Gutierrez P., Villalon L., Losa J. E., Henriquez-Camacho C. (2014). Treatment of febrile neutropenia and prophylaxis in hematologic malignancies: A critical review and update. *Advances in Hematology*.

[2] Freifeld A. G., Bow E. J., Sepkowitz K. A. (2011). Executive summary: clinical practice guideline for the use of antimicrobial agents in neutropenic patients with cancer: 2010 update by the Infectious Diseases Society of America. *Clinical Infectious Diseases*.

[3] Gea-Banacloche J. (2013). Evidence-based approach to treatment of febrile neutropenia in hematologic malignancies. *Hematology / the Education Program of the American Society of Hematology. American Society of Hematology. Education Program*.

[4] Safdar A. (2011). *Principles and Practice of Cancer Infectious Diseases*.

[5] Bhardwaj A. S., Navada S. C. (2015). Management of chemotherapy-induced neutropenic fever. *Hospital Practice*.

[6] Pagano L., Caira M., Nosari A. (2011). The use and efficacy of empirical versus pre-emptive therapy in the management of fungal infections: the HEMA e-Chart project. *Haematologica*.

